# Lipidomic Analysis Reveals the Protection Mechanism of GLP-1 Analogue Dulaglutide on High-Fat Diet-Induced Chronic Kidney Disease in Mice

**DOI:** 10.3389/fphar.2021.777395

**Published:** 2022-03-01

**Authors:** Martin Ho Yin Yeung, Ka Long Leung, Lai Yuen Choi, Jung Sun Yoo, Susan Yung, Pui-Kin So, Chi-Ming Wong

**Affiliations:** ^1^ Department of Health Technology and Informatics, The Hong Kong Polytechnic University, Kowloon, Hong Kong SAR, China; ^2^ Department of Medicine, The University of Hong Kong, Pokfulam, Hong Kong SAR, China; ^3^ University Research Facility in Life Sciences, The Hong Kong Polytechnic University, Kowloon, Hong Kong SAR, China

**Keywords:** lipidomics, mass spectrometry imaging, obesity, chronic kidney disease, diabetic kidney disease, GLP-1R agonists, dulaglutide

## Abstract

Many clinical studies have suggested that glucagon-like peptide-1 receptor agonists (GLP-1RAs) have renoprotective properties by ameliorating albuminuria and increasing glomerular filtration rate in patients with type 2 diabetes mellitus (T2DM) and chronic kidney disease (CKD) by lowering ectopic lipid accumulation in the kidney. However, the mechanism of GLP-1RAs was hitherto unknown. Here, we conducted an unbiased lipidomic analysis using ultra-high-performance liquid chromatography/electrospray ionization-quadrupole time-of-flight mass spectrometry (UHPLC/ESI-Q-TOF-MS) and matrix-assisted laser desorption/ionization mass spectrometry imaging (MALDI-MSI) to reveal the changes of lipid composition and distribution in the kidneys of high-fat diet-fed mice after treatment with a long-acting GLP-1RA dulaglutide for 4 weeks. Treatment of dulaglutide dramatically improved hyperglycemia and albuminuria, but there was no substantial improvement in dyslipidemia and ectopic lipid accumulation in the kidney as compared with controls. Intriguingly, treatment of dulaglutide increases the level of an essential phospholipid constituent of inner mitochondrial membrane cardiolipin at the cortex region of the kidneys by inducing the expression of key cardiolipin biosynthesis enzymes. Previous studies demonstrated that lowered renal cardiolipin level impairs kidney function via mitochondrial damage. Our untargeted lipidomic analysis presents evidence for a new mechanism of how GLP-1RAs stimulate mitochondrial bioenergetics via increasing cardiolipin level and provides new insights into the therapeutic potential of GLP-1RAs in mitochondrial-related diseases.

## Introduction

Diabetic kidney disease (DKD) is one of the most serious progressive complications commonly observed in humans with diabetes (Yu and Bonventre, 2018). Albuminuria is a primary but not essential sign of kidney function decline in DKD (Ritz et al., 2011; Pugliese et al., 2020). Progression of DKD further alters metabolic dysregulation and hemodynamic disorder, leading to glomerular hypertension, ischemia, increased oxidative stress, and an upregulation of the renin–aldosterone system, which reduced the estimated glomerular filtration rate (eGFR) (Yamazaki et al., 2021). Abnormal histopathological changes including thickening of the glomerular basement membrane, enlargement of the glomeruli area, mesangial expansion, and glomerulosclerosis are observed in DKD (Yamazaki et al., 2021). If not adequately treated, kidney function will gradually decline. Up to 50% of DKD cases will result in a devastating medical problem—end-stage renal disease (ESRD) (Tuttle et al., 2014). ESRD patients need regular long-term dialysis or kidney transplant to maintain their life. Although the incidence rate of DKD in diabetic patients has slightly decreased in recent years, because the number of diabetic patients kept increasing, the prevalence of DKD-induced ESRD remains high (Alicic et al., 2017; Jitraknatee et al., 2020).

As hypertension is prevalent in most patients with DKD, the common therapeutic strategy for DKD is lowering their blood pressures (Ritz et al., 2011; Kitada et al., 2014). Anti-hypertensive agents such as renin–angiotensin–aldosterone system (RAS) inhibitors, angiotensin-converting enzyme (ACE) inhibitors, and angiotensin II receptor blockers (ARB) are used to treat DKD (Lewis et al., 1993; Brenner et al., 2001; Lewis et al., 2001; Yacoub and Campbell, 2015). Unfortunately, patients treated with RAS inhibitors have a definite increase in the chances of hyperkalemia and hypotension and still have a high risk of ESRD (Burns and Cherney, 2019; Zhang et al., 2020). The efficacy and safety of ACE inhibitors and ARB in DKD patients are still controversial (Wang et al., 2018; He et al., 2020). Because DKD is associated with a widely heterogeneous range of pathological features, such as the progression of DKD, which can be either classical albuminuric or new non-albuminuric pathways (Pugliese et al., 2020), different therapeutic strategies for the treatment of DKD are required.

It is widely accepted that chronic hyperglycemia plays a key role in triggering oxidative stress and inflammation to promote the progression of DKD (Amorim et al., 2019; DeFronzo et al., 2021). However, the increase in the use of diabetes-related medications in the last decades seems to not have reduced the prevalence of DKD (de Boer et al., 2011; Afkarian et al., 2016; Kume et al., 2019). In addition, due to the potential danger of hypoglycemia in patients with diabetes and impaired renal function (Pugliese et al., 2020), many diabetic drugs such as metformin is not recommended for DKD patients with a low eGFR (Kawanami et al., 2020). Safe and effective therapies are an urgent need to prevent DKD progression and worsening. Until recently, accumulating evidence from clinical and experimental studies demonstrated that the glycemic control drugs, glucagon-like-peptide-1 receptor agonists (GLP-1RAs), are effective and safe glycemic control drugs for diabetic patients with kidney disease (Edwards and Minze, 2015; Coskun et al., 2018). Unexpectedly, the exploratory renal outcomes from GLP-1RA cardiovascular outcome trials suggested that GLP-1RAs have unexpected renoprotective properties (Yin et al., 2020).

GLP-1RAs mimic the structure and function of glucagon-like peptide-1 (GLP-1). GLP-1 is secreted from L cells in the gut postprandial to stimulate insulin secretion from the pancreatic islets in response to elevated glucose levels after a meal (Meloni et al., 2013). In addition to its glucose-lowering effect, GLP-1 also regulates appetite, gut motility, and lipid metabolism (Campbell and Drucker, 2013). As the endogenous GLP-1 is reduced in diabetic patients, GLP-1-based therapies are developed for glycemic control and weight loss in patients with type 2 diabetes. Interestingly, recent multiple randomized clinical trials (RCTs) have reported that GLP-1RAs significantly decrease in the secondary renal outcomes. For example, GLP-1RAs dulaglutide and lixisenatide reduced the development and progression of macroalbuminuria with modest effects on eGFR (Muskiet et al., 2018; Tuttle et al., 2018; Gerstein et al., 2019). Several ongoing RCTs of GLP-1RAs in primary kidney outcome are going to validate the findings (Gorriz et al., 2020).

Many mechanisms for GLP-1RA renoprotection were suggested. For example, GLP-1RAs reduced glycation end products, leading to lowered inflammation in renal mesangial cells (Chang et al., 2017). GLP-1RAs modulate kidney sodium homeostasis via NHE3 activity (Carraro-Lacroix et al., 2009) and lower circulating RAS concentrations (Skov, 2014), reducing oxidative stress (Rodriguez et al., 2020) and decreasing ectopic lipid accumulation (Wang et al., 2018; Guo et al., 2018). Among them, lipotoxicity in the progression of DKD has gained considerable attention in recent years (Nicholson et al., 2020; Kim et al., 2021). Lipid droplets were found in the glomerular and tubular portion of DKD patients (Thongnak et al., 2020). Reducing renal lipotoxicity can inhibit the development of DKD-associated pathologies by re-sensitizing podocytes to insulin signaling in DKD mouse models (Falkevall et al., 2017). However, it remains unclear whether these renoprotective properties are a direct effect of GLP-1RAs on kidney lipid metabolism or an indirect effect of improvements in overall metabolic syndromes. Thus, further studies are required by elucidating new mechanisms for the study of the relationship between GLP-1RAs and DKD.

With the advancement in mass spectrometry technology (Ellis et al., 2007; Nam et al., 2015; Liu et al., 2019), we used untargeted lipidomic approaches to comprehensively unravel the changes in lipid contents of the diet-induced kidney damage from obese mice after treatment of a GLP-1RA dulaglutide and hence to explore the underlying mechanisms for its renoprotective effect. In this report, we presented the evidence that dulaglutide protects renal function by elevating the level of a unique mitochondrial inner membrane phospholipid, cardiolipin, in the kidney cortex region via the upregulation of the mRNA expression of cardiolipin synthesis enzymes, which leads to alleviation of overnutrition-impaired kidney mitochondrial bioenergetics.

## Materials and Methods

### Animal Study and Sample Collection

Male C57BL/6 mice were purchased from The Hong Kong Polytechnic University. The animal study and treatment procedures were approved by our Animal Ethics Committee. All mice were housed at a temperature of 25 ± 2°C and humidity of 60 ± 5% with a 12 h light and dark cycle and were given *ad libitum* access to water and diet. The 8-week-old mice were divided randomly into three subgroups: standard chow control (STC), high-fat diet-induced diabetic nephropathy (HFD), and high-fat diet-induced diabetic nephropathy with dulaglutide treatment (H + Dula). The STC group was fed a standard chow (13.2% fat, 24.7% protein, and 62.1% carbohydrates; PicoLab Rodent Diet 20 #5053, St. Louis, United States), and others were fed with a high-fat diet (60% fat, 20% protein, and 20% carbohydrates; Research Diets Inc., New Brunswick, United States) for 12 weeks. Then, the treatment group was intraperitoneally administrated with dulaglutide in the morning at a dose of 0.6 mg/kg body weight (BW) once every 7 days and lasting for 4 weeks. An equal volume of saline was also injected into mice in the STC and HFD groups.

The full body composition of mice was analyzed using a Minispec LF-50 nuclear magnetic resonance spectrometer according to the manufacturer’s protocol (Bruker, Germany). The blood glucose of the mice was measured from the tail vein using the Accu-Chek^®^ glucometer (Roche Diagnostics, Indiana, United States). The 24 h urine volume was measured and collected before week 19 and after week 24 of dulaglutide treatment by individual metabolic cages. Urine samples were centrifuged at 2,000 × g for 10 min at 4°C, aliquoted, and stored at −80°C before analysis. Albumin concentration was determined using the mouse albumin ELISA (ab207620, Abcam, United Kingdom) kit according to the manufacturer’s instructions. Serum urea level was determined using the QuantiChrom Urea Assay Kit (BioAssay Systems, United States), and blood urea nitrogen (BUN) level was calculated. For intraperitoneal glucose tolerance test (IPGTT), the mice were then fasted overnight for 16 h with *ad libitum* access to water only. Basal tail vein fasting blood glucose was obtained and measured before intraperitoneal injection of glucose solution at a dose of 1.5 g/kg/mouse. After the completion of high-fat diet feeding and dulaglutide treatment, mice were fasted overnight for 16 h and were anesthetized for blood collection by cardiac puncture. Metabolic organs including the kidneys and liver were harvested and separated for snap-freezing in liquid nitrogen or formalin-fixed for subsequent analyses.

### Determination of Renal Histopathology

Tissues were isolated and fixed for 24 h in 10% neutral-buffered formalin for subsequent tissue processing and embedded in paraffin (FFPE). The FFPE sections of 4 μm thickness were prepared and mounted on uncoated glass slides for hematoxylin and eosin (H&E) and periodic acid Schiff–methenamine silver (PASM) staining. Oil Red O (ORO) staining for neutral lipid was performed on snap-frozen tissues. Tissues were trimmed and cryosectioned using a CryoStar NX70 cryostat (Thermo Scientific, United States) at 8 μm thickness and then thaw-mounted on uncoated glass slides. They were dried for 5 min and fixed in 4% neutral-buffered formalin before proceeding with ORO staining according to the manufacturer’s protocol (BioGnost, Croatia). Additional kidney cryosections were thaw-mounted on indium tin oxide (ITO) slides (Bruker, Germany). The slides were dried under vacuum in a desiccator for approximately 30 min and stored at −80°C until use within 7 days.

### Tissue Lipid Extraction With Quality Control Sample Preparation

The tissue samples were thawed for 30 min at 4°C. Lipids were extracted according to Bligh and Dyer’s (1959) method. Briefly, 200 μl of ice-cold methanol was added to each sample, vortexed for 15 s, and sonicated in a pre-cooled sonicator for 15 min. Then, 200 and 100 μl of ice-cold chloroform and water were added, respectively, into each sample, vortexed for 15 s, and sonicated for another 15 min. The resulting mixture was centrifuged at 10,000 RPM for 5 min at 4°C. The lower phase containing lipids was separated and dried using a SpeedVac concentrator (Labconco, MO, United States) prior to storage at −80°C. Before Ultra-high-performance liquid chromatography/electrospray ionization-quadrupole time-of-flight mass spectrometry (UHPLC/ESI-QTOF-MS) analysis, the lipid fractions were reconstituted in 100 μl of ice-cold methanol and chloroform (1:1, v/v), followed by sonication for 15 min. Samples containing 10 μl of lipids were pooled and vortexed to form the quality control (QC) samples.

### UHPLC/ESI-QTOF-MS Analysis

UHPLC/ESI-QTOF-MS analysis was performed in both positive and negative ionization modes with an Agilent 6540B ESI-QTOF-MS connected with an Agilent 1290 UHPLC (Agilent, United States). Liquid chromatography was carried out using a Waters ACQUITY BEH C18 column. The mobile-phase solvents consisted of A (60:40 acetonitrile:water (v/v) with 5 mM ammonium formate and 0.1% (v/v) formic acid) and B (90:10 isopropanol:acetonitrile (v/v) with 5 mM ammonium formate and 0.1% (v/v) formic acid). Chromatographic separation was performed at a flow rate of 0.3 ml/min with an elution gradient. Column and sample chamber temperatures were set at 55°C and 4°C, respectively.

### Multivariate Statistical Analysis

Raw LCMS data collected were subjected to peak picking, alignment, and normalization (by all compound abundance) by the Progenesis QI software (Nonlinear Dynamics-Waters, Milford, United States), from which a dataset consisting of retention time, mass-to-charge ratio (*m/z*)*,* and normalized abundance was generated. The dataset was further processed by the EZInfo 2.0 software (Sartorius, Germany) for multivariate data analysis, including principal component analysis (PCA) and orthogonal partial least-squared discriminant analysis (OPLS-DA). OPLS-DA was used to create the variable importance in projection (VIP) plot. Compounds with a VIP value >1 and *t*-test with *p* < 0.05 were considered as potential differential markers, which were identified with the Human Metabolome database and LIPID MAPS Structure database (LIPID MAPS Lipidomics Gateway) based on accurate molecular mass and fragment ion pattern.

### MALDI-MSI Analysis

A matrix of 10 mg/ml of 2,5-dihydroxybenzoic acid (DHB) was evenly sprayed onto the ITO slide using an HTX TM-Sprayer (HTX Imaging, North Carolina, United States). The homogeneity of the matrix layer was assessed using a light inverted microscope. A peptide calibration standard (Bruker, Germany) was then applied to the slide for mass calibration. Matrix-assisted laser desorption/ionization mass spectrometry imaging (MALDI-MSI) analysis was performed with a Bruker UltrafleXtreme matrix-assisted laser desorption/ionization time-of-flight/time-of-flight mass spectrometer equipped with a 355 nm smart beam laser source (Bruker, Germany). MALDI-MSI data were acquired with the Bruker flexImaging program. The spatial resolution was set at 100 × 100 μm, and mass spectra were collected with a *m*/*z* range of 400–2,000 and 500 consecutive laser shots accumulated per pixel. Data analysis was performed with the SCiLS Lab software version 2020b Pro (Bruker, Germany). Root-mean-square normalization was applied to normalize the ion intensity during ion image generation. Lipids were identified with the LIPID MAPS (LIPID MAPS Lipidomics Gateway) database based on accurate molecular mass and fragment ion pattern.

### Reverse Transcription-Quantitative Polymerase Chain Reaction

Total RNAs were extracted from kidney tissue samples using RNAiso Plus (Takara, China) according to manufacturer’s instruction and were quantified using a NanoDrop One spectrophotometer (Thermo Scientific, United States). First-strand complementary DNA (cDNA) synthesis was carried out from 1 μg RNA using PrimeScript RT Kit (Takara, China). TB Green Premix Ex Taq was incorporated with cDNA and primers for subsequent amplification and fluorescent detection. Amplification was performed using LightCycler^®^ 96 (Roche, Germany). Relative gene expression was calculated using the 2^−ΔΔCt^ method. Supplementary Table S1 shows the primer sequences used in this study.

### Statistical Analysis

Statistical analysis was performed routinely using GraphPad Prism 8 (GraphPad Software, San Diego, CA), with *p* < 0.05 being considered as significant. All values were expressed as mean ± standard error of the mean (SEM). For comparisons among groups, one-way ANOVA was performed with Tukey post-hoc test with the HFD group set as the reference group to compare multiple groups. Each experiment was repeated at least three times.

## Results

### Dulaglutide Can Attenuate the Progression of High-Fat Diet-Induced Kidney Injury in Mice

**FIGURE 1:**
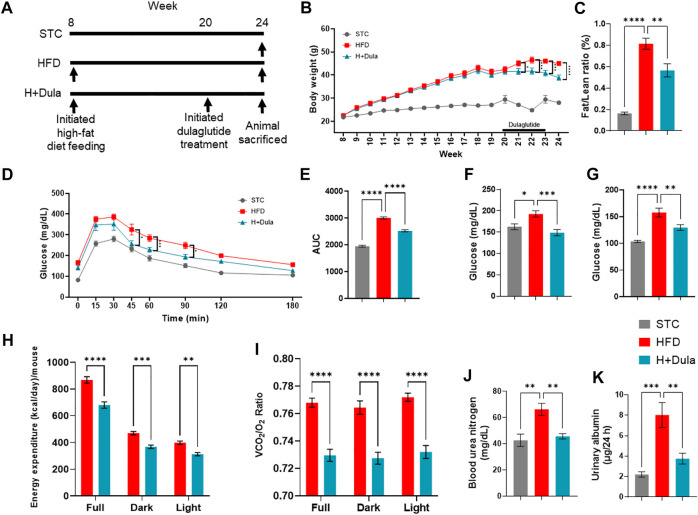
Dulaglutide improves high-fat diet-induced kidney damage in mice. **(A)** Timeline of high-fat diet feeding and dulaglutide treatment. **(B)** Changes in body weight on STC, HFD and H + Dula group of mice (One-way ANOVA with Tukey post-hoc test was performed between the HFD and H + Dula groups; *p < 0.05, ***p < 0.001 and ****p < 0.0001 compared between HFD and H + Dula groups). **(C)** ^1^H nuclear magnetic resonance was used to calculate fat/lean ratio (week 24). **(D)** Intraperitoneal glucose tolerance test (IPGTT) was performed (One-way ANOVA with Tukey post-hoc test was performed between the HFD and H + Dula groups* *p* < 0.05 and ***p < 0.001) and **(E)** area under curve was calculated (week 22). **(F)** Fed and **(G)** fasting glucose were measured (week 24). **(H)** Energy expenditure and **(I)** respiratory exchange ratio (RER) was calculated by volume of carbon dioxide over volume of oxygen over 24 h (Supplementary Figure S1). **(J)** Serum urea level was determined and blood urea nitrogen (BUN) calculated. **(K)** 24 h urine was collected and albuminuria determined. Data represents means ± SEM, *n* = 6–9 mice per group. *p < 0.05, **p < 0.01, ***p < 0.001 and ****p < 0.0001.

Previous studies demonstrated that GLP-1RAs could be utilized in treating DKD in humans (Marso et al., 2016a; Marso et al., 2016b; Muskiet et al., 2018; Tuttle et al., 2018; Gerstein et al., 2019). To explore the mechanism, we used high-fat diet to promote renal injury in mice (Figure 1A). As previously reported (Findeisen et al., 2019; Park et al., 2019; Svendsen et al., 2020; Hupa-Breier et al., 2021), treatment of dulaglutide lowered the body weight (Figure 1B) by reducing the fat mass (Figure 1C and Supplementary Figures S1A−F), improved glucose homeostasis (Figures 1D–G), and reduced the energy expenditure (Figure 1H), respiratory exchange ratio (Figure 1I), and food intake (Supplementary Figure S1G). Importantly, dulaglutide improved kidney function in the H + Dula group mice and reduced BUN level (Figure 1J) and albuminuria (Figure 1K) compared with the HFD group.

### Dulaglutide Attenuates Renal Tissue Pathological Changes in High-Fat Diet-Fed Mice

**FIGURE 2:**
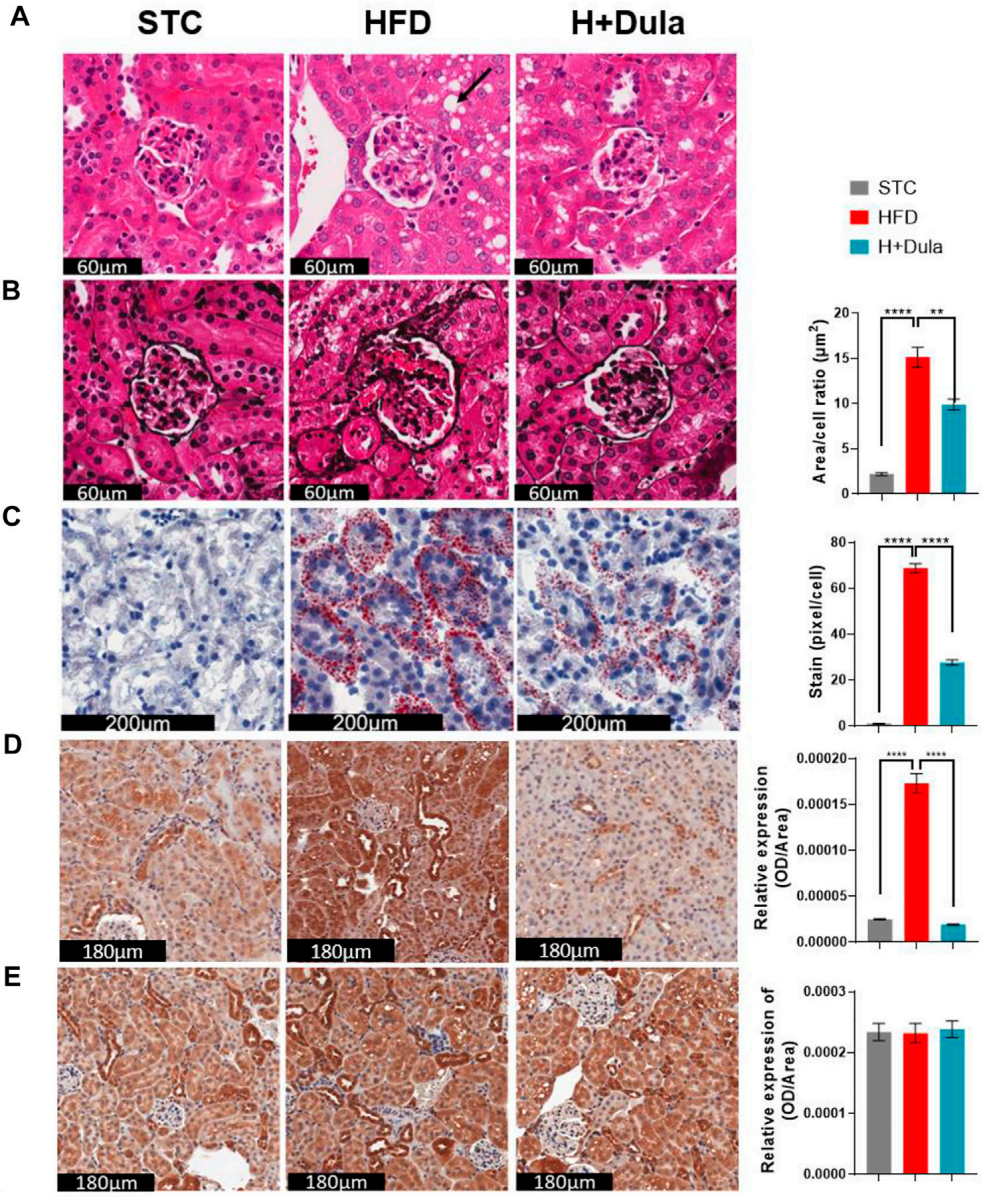
Dulaglutide improves high-fat diet-induced renal morphological changes. **(A)** Haematoxylin and Eosin (H&E) stain show cytoplasmic vacuole formation in the renal tubules (black arrow) was observed. **(B)** Periodic Schiff-Methenamine (PASM) was stained to assess for increasing mesangial expansion. The area per cell of glomeruli were also quantified Oil Red O staining was performed to visualize neutral lipids at cortex **(C)** and medulla **(D)** region. Immunohistochemistry of **(E)** kidney injury molecule-1 (KIM-1) and **(F)** glucagon-like peptide-1 receptor (GLP-1R) was performed. Image analysis was performed using ImageJ software, and the average area of glomeruli was calculated. The average staining of neutral lipids were quantified by red stained pixel/cell. Optical density of renal tubules was calculated for the relative expression of KIM-1 and GLP-1R in the renal tubules. All image analysis was calculated from 30 random area from each mouse. The representative images of all stains are from three different groups. Data represents means ± SEM, *n* = 6–9 mice per group. **p < 0.01 and ****p < 0.0001.

We also performed histological examination and revealed that the kidney of high-fat diet-fed mice developed vacuolization of renal tubules, same as that observed in obese patients with DKD (Figure 2A, black arrow) (Liptak and Ivanyi, 2006; Watanabe et al., 2021). Reduction in the area of vacuolization was observed in the H + Dula group as compared to the HFD group (Figure 2A). PASM staining showed mesangial expansion, enlargement of the glomerular tuft, and thickening of the tubular basement membrane in the HFD group as compared with the STC group, and the area showed a significant reduction in size after dulaglutide treatment (Figure 2B).

To visualize the renal neutral lipid, ORO staining was used. A dramatic increase in lipids per cell in the HFD group as compared to the STC control was observed mainly in the cortex region of the kidney. The H + Dula group showed a significant reduction in the amount of lipid droplet per cell as compared with HFD group, but the lipid level was still much higher than that of the STC group (Figure 2C). High-fat diet also increased lipid accumulation within the medulla region but not as predominate as in the cortex region (Figure 2D). To assess for renal tubular injury in the kidney, immunohistochemical staining (IHC) was performed with kidney injury molecule-1 (KIM-1; Figure 2E). KIM-1 is highly expressed in renal tubules in the HFD group as compared with the STC group (*p* < 0.0001). A significant reduction of KIM-1 expression in the H + Dula group is found, consistent with previous urinary albumin data, indicating reduced damage in renal tubules after dulaglutide treatment. These results suggest that dulaglutide treatment attenuated the progression of high-fat diet-induced kidney damage in our mouse model. As previous study reported renal tubular GLP-1R expression is reduced in chronic kidney disease (Choi et al., 2019), IHC staining of kidney sections was performed to explore GLP-1R protein expression. In brief, there were no significant changes in GLP-1R expression after high-fat diet and dulaglutide treatment in the renal tubules (Figure 2F).

### Diverse Alterations in the Kidney Lipidomes of Dulaglutide-Treated High-Fat Diet-Fed Mice

**FIGURE 3:**
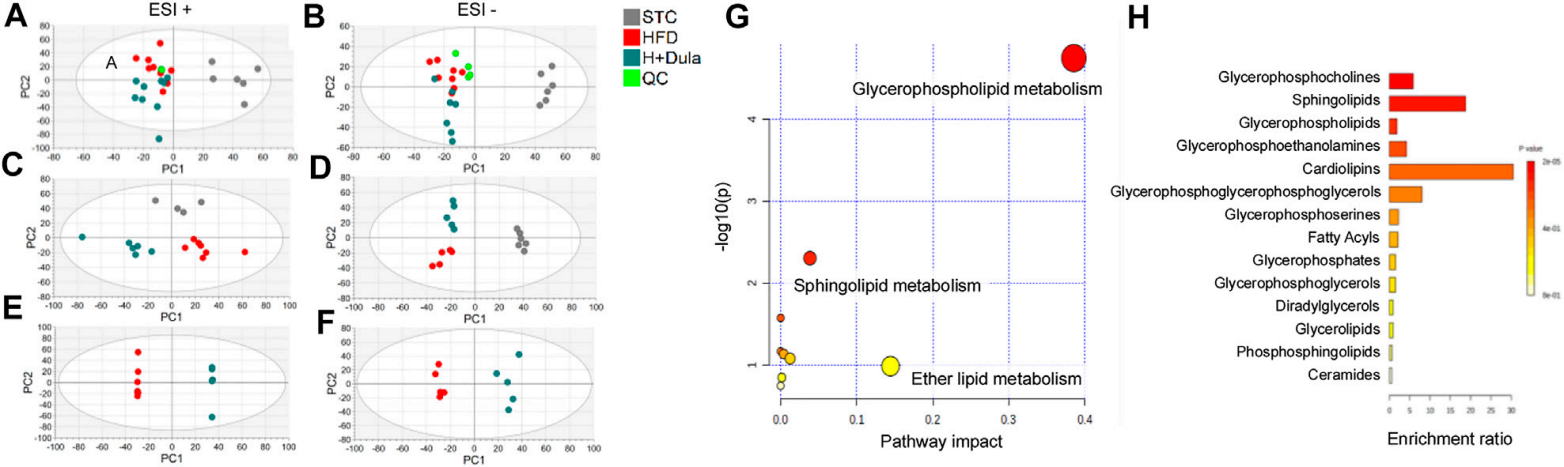
Bioinformatics analysis of lipidomics of the kidneys of STC, HFD and H + Dula groups **(A–F)** The score plots from PCA, PLS-DA and OPLS-DA model between the HFD and the H + Dula group. UHPLC/ESI-QTOF-MS in positive and negative electrospray ionization mode were used. **(A,B)** PCA score plot, **(C,D)** PLS-DA score plot and **(E,F)** OPLS-DA score plot of lipids from UHPLC/ESI-QTOF-MS in positive and negative mode, respectively. Positive PLS-DA score plot: R^2^X_(cum)_ = 0.534, R^2^Y_(cum)_ = 0.990, Q^2^_(cum)_ = 0.792; negative PLS-DA score plot: R^2^X_(cum)_ = 0.561, R^2^Y_(cum)_ = 0.990, Q^2^_(cum)_ = 0.885. Positive OPLS-DA score plot: R^2^X_(cum)_ = 0.563, R^2^Y_(cum)_ = 0.999, Q^2^_(cum)_ = 0.706; negative OPLS-DA score plot: R^2^X_(cum)_ = 0.414, R^2^Y_(cum)_ = 0.975, Q^2^_(cum)_ = 0.614. **(G)** Summary of renal lipidomic pathways of high-fat diet-fed mice altered by dulaglutide treatment. Each point represents one lipidomic pathway. The size of dot and the shade of color are positively related to the effect on the lipidomic pathway. **(H)** Lipid sets enrichment overview of the kidneys of high-fat diet-fed mice after treatment with dulaglutide.

To explore the changes in lipidomes of kidney in high-fat diet-fed mice after dulaglutide treatment, UHPLC/ESI-QTOF-MS analysis was performed. The general visualization of interrelations and separation among the STC control, HFD, and H + Dula groups was plotted (Figure 3). PCA score plots show that the lipidomic profiles of the HFD and H + Dula groups are clustered and separated from the STC control group (Figures 3A,B). To further facilitate the detection and differentiation of potential compounds of interest among the three groups, PLS-DA was plotted. PLS-DA shows a clear separation among all three groups (Figures 3C,D). In addition, an OPLS-DA analysis was performed on mass spectra and normalized abundance obtained from the HFD and H + Dula groups to show a significant separation between these two groups (Figures 3E,F). These results suggest that 4 week dulaglutide treatment dramatically changed the lipid species in the kidneys of high-fat diet-fed mice.

### Pathway and Enrichment Analysis of Lipids in the Kidney of High-Fat Diet-Fed Mice After Dulaglutide Treatment

Pathway analysis was performed and identified several altered metabolic pathways in the kidneys (Figure 3G). The top altered pathway is glycerophospholipid metabolism. Previous studies demonstrated that the composition and abundance of glycerophospholipids are changed in the serum and muscle of obese mice with T2DM (Rauschert et al., 2016; Wolfgang, 2021). As glycerophospholipids can undergo esterification with amino acids to form a range of glycerophospholipid subtypes, unbiased lipid set enrichment analysis was performed to screen for differently expressed lipids. Intriguingly, an essential constituent of the inner mitochondrial membrane lipid, cardiolipin, shows the highest enrichment (Figure 3H). As the above analyses did not provide any information for their relative levels, we further analyzed the changes in the level of each lipid species in the HFD and H + Dula groups. In short, we identified 62 lipids of interest and listed their details in Table 1. In brief, there was reduced abundance of most identified species for diacylglycerols (DG), phosphatidic acids (PA), phosphatidylglycerols (PG), and triglycerides (TG) in the kidney of the H + Dula group. Unexpectedly, the levels of a number of lipid species were remarkably increased in the H + Dula group, such as all identified ceramides (Cer), four out of five cardiolipin species (CL), and eight out of nine phosphatidylethanolamine species (PE).

**TABLE 1:** Identified differential lipids between HFD model over H + Dula model in kidney tissue obtained by UHPLC/ESI-QTOF-MS in ESI positive and negative mode.

NO.	Abbreviation	Molecular formula	+/−	Adduct form	Mass-to-charge ratio (m/z)	Retention time (min)	Mass error (ppm)	*p*-value	Fold change (log_2_FC)
1	Cer(d42:1)	C_42_H_83_NO_3_	+	M + H	650.646	11.42	2.15	0.0133	0.244
2	Cer(d40:2)	C_40_H_77_NO_3_	−	M + FA-H	664.585	9.36	0.66	0.0428	0.160
3	CL (72:0)	C_81_H_158_O_17_P_2_	−	M-H	1,464.083	7.03	−4.72	0.0420	0.970
4	CL (74:3)	C_83_H_156_O_17_P_2_	−	M-H	1,486.079	7.85	2.89	0.0277	0.369
5	CL (76:1)	C_85_H_164_O_17_P_2_	−	M + FA-H	1,564.146	7.69	2.02	0.0371	−0.210
6	CL (76:12)	C_85_H_142_O_17_P_2_	−	M-H	1,495.962	11.37	−2.10	0.0487	0.321
7	CL (78:2)	C_87_H_166_O_17_P_2_	−	M + Cl	1,580.130	7.04	0.54	0.0433	0.240
8	DG (32:5)	C_35_H_58_O_5_	+	M + H	559.435	6.55	−1.97	0.0245	−0.497
9	DG (42:8)	C_45_H_72_O_5_	+	M + H	693.544	9.07	−1.98	0.0048	−0.638
10	DG (38:5)	C_41_H_70_O_5_	+	M + H	643.529	9.23	−1.38	0.0014	−0.348
11	DG (40:6)	C_43_H_72_O_5_	+	M + NH_4_	686.570	8.81	−2.77	0.0194	0.525
12	DG (42:7)	C_45_H_74_O_5_	+	M + H	695.560	9.80	−0.91	0.0488	−0.939
13	DG (42:9)	C_45_H_70_O_5_	+	M + H	691.528	8.48	−1.82	0.0084	−0.817
14	Glu-Cer(d30:1)	C_36_H_69_NO_8_	+	M + H	644.510	5.66	1.39	0.0212	−0.196
15	LysoPC(O-18:0)	C_26_H_56_NO_6_P	+	M + H	510.391	3.90	−1.61	0.0254	0.680
16	LysoPE (22:0)	C_27_H_56_NO_7_P	+	M + H	538.389	3.78	4.73	0.0391	−0.667
17	PA (33:2)	C_36_H_67_O_8_P	+	M + H	659.467	6.27	3.69	0.0322	−0.937
18	PA (41:0)	C_44_H_87_O_8_P	+	M + H	775.623	11.49	1.81	0.0217	−1.658
19	PC(34:2)	C_42_H_80_NO_8_P	+	M + H	758.572	6.96	3.12	0.0097	0.360
20	PC(40:5)	C_48_H_86_NO_8_P	+	M + Na	858.596	8.04	−2.85	0.0489	0.652
21	PC(42:10)	C_50_H_80_NO_8_P	−	M-H	852.558	4.72	3.38	0.0064	−0.596
22	PC(P-38:5)	C_46_H_82_NO_7_P	−	M + Cl	826.554	6.01	2.56	0.0199	0.545
23	PC(P-40:4)	C_48_H_88_NO_7_P	+	M + Na	844.621	7.08	2.25	0.0127	0.466
24	PC(40:5)	C_48_H_86_NO_8_P	+	M + H	836.617	6.98	0.22	0.0271	−0.719
25	PC(P-38:5)	C_46_H_82_NO_7_P	+	M + H	792.591	6.94	0.86	0.0179	−0.362
26	PC(P-40:5)	C_48_H_86_NO_7_P	+	M + H	820.622	7.83	1.21	0.0195	−0.633
27	PC(42:10)	C_50_H_80_NO_8_P	+	M + H	854.570	5.51	0.93	0.0487	0.502
28	PC(o-38:0)	C_46_H_94_NO_7_P	+	M + H	804.684	10.95	−0.25	0.0093	1.297
29	PC(o-44:4)	C_52_H_98_NO_7_P	+	M + H	880.719	12.17	4.06	0.0263	1.024
30	PC(P-36:0)	C_44_H_88_NO_7_P	+	M + Na	796.620	8.28	1.35	0.0020	0.402
31	PC(P-38:4)	C_46_H_84_NO_7_P	+	M + H	794.605	7.21	−1.23	0.0260	−0.470
32	PC(P-40:6)	C_48_H_84_NO_7_P	+	M + H	818.605	6.88	−0.87	0.0289	−0.356
33	PC(P-38:1)	C_46_H_90_NO_7_P	+	M + Na	822.637	8.16	3.12	0.0111	−0.533
34	PE (34:2)	C_39_H_74_NO_8_P	+	M + H	716.523	7.23	0.11	0.0055	0.264
35	PE (P-34:2)	C_39_H_74_NO_7_P	−	M + Cl	734.492	6.63	3.94	0.0206	−0.350
36	PE (38:5)	C_43_H_76_NO_8_P	−	M-H	764.520	7.03	−4.10	0.0417	0.210
37	PE (38:0)	C_43_H_86_NO_8_P	+	M + H	776.614	9.73	−3.00	0.0408	0.721
38	PE (40:8)	C_45_H_74_NO_8_P	+	M + H	788.524	8.19	2.36	0.0086	0.790
39	PE (P-36:2)	C_41_H_78_NO_7_P	+	M + H	728.559	8.65	−0.02	0.0281	0.427
40	PE (P-38:4)	C_43_H_78_NO_7_P	−	M + Cl	786.524	6.81	3.79	0.0082	0.194
41	PE (P-42:2)	C_47_H_90_NO_7_P	+	M + H	812.656	13.70	3.54	0.0263	1.318
42	PE-NMe(32:5)	C_38_H_66_NO_8_P	+	M + H	696.463	3.90	4.83	0.0260	0.728
43	PG (36:3)	C_42_H_77_O_10_P	−	M-H	771.517	5.51	−0.94	0.0333	−0.393
44	PG (34:2)	C_40_H_75_O_10_P	−	M-H	745.502	5.45	−0.67	0.0148	−0.891
45	PG (36:2)	C_42_H_79_O_10_P	−	M-H	773.533	6.09	−1.09	0.0231	−0.354
46	PG (38:6)	C_44_H_75_O_10_P	−	M-H	793.499	4.73	−4.45	0.0164	−0.348
47	PG (36:4)	C_42_H_75_O_10_P	−	M-H	769.503	5.46	0.08	0.0451	0.685
48	PI(38:3)	C_47_H_85_O_13_P	+	M + H	889.580	7.07	−0.34	0.0466	0.558
49	PI(40:5)	C_49_H_85_O_13_P	−	M-H	911.564	6.53	−1.42	0.0406	−0.420
50	PS(P-28:0)	C_34_H_66_NO_9_P	+	M + Na	686.439	2.72	4.03	0.0403	0.961
51	PS(37:1)	C_43_H_82_NO_10_P	+	M + H	804.572	6.51	−3.89	0.0067	−0.325
52	PS(32:0)	C_38_H_74_NO_10_P	−	M-H	734.499	6.79	2.25	0.0359	−0.610
53	SM(d36:1)	C_41_H_83_N_2_O_6_P	+	M + H	731.608	7.79	1.92	0.0229	−0.222
54	SM(d36:2)	C_41_H_81_N_2_O_6_P	+	M + K	767.544	8.17	−3.85	0.0036	0.478
55	TG (48:4)	C_51_H_90_O_6_	+	M + H	799.681	11.65	0.02	0.0093	0.986
56	TG (52:6)	C_55_H_94_O_6_	+	M + NH_4_	868.739	12.67	0.20	0.0240	−0.399
57	TG (54:9)	C_57_H_92_O_6_	+	M + NH_4_	890.724	11.83	1.09	0.0184	−0.741
58	TG (49:2)	C_52_H_96_O_6_	+	M + Na	839.710	13.61	−0.35	0.0363	−0.457
59	TG (50:5)	C_53_H_92_O_6_	+	M + Na	847.678	11.97	−0.68	0.0370	−0.941
60	TG (60:12)	C_63_H_98_O_6_	+	M + NH_4_	968.770	12.17	−0.08	0.0011	−0.782
61	TG (62:13)	C_65_H_100_O_6_	+	M + NH_4_	994.785	12.14	−0.51	0.0017	−1.237
62	TG (62:14)	C_65_H_98_O_6_	+	M + NH_4_	992.770	11.51	−0.24	0.0057	−1.735

### Dulaglutide Treatment Increases the Level of Renal Cardiolipin in the Cortex Region

**FIGURE 4:**
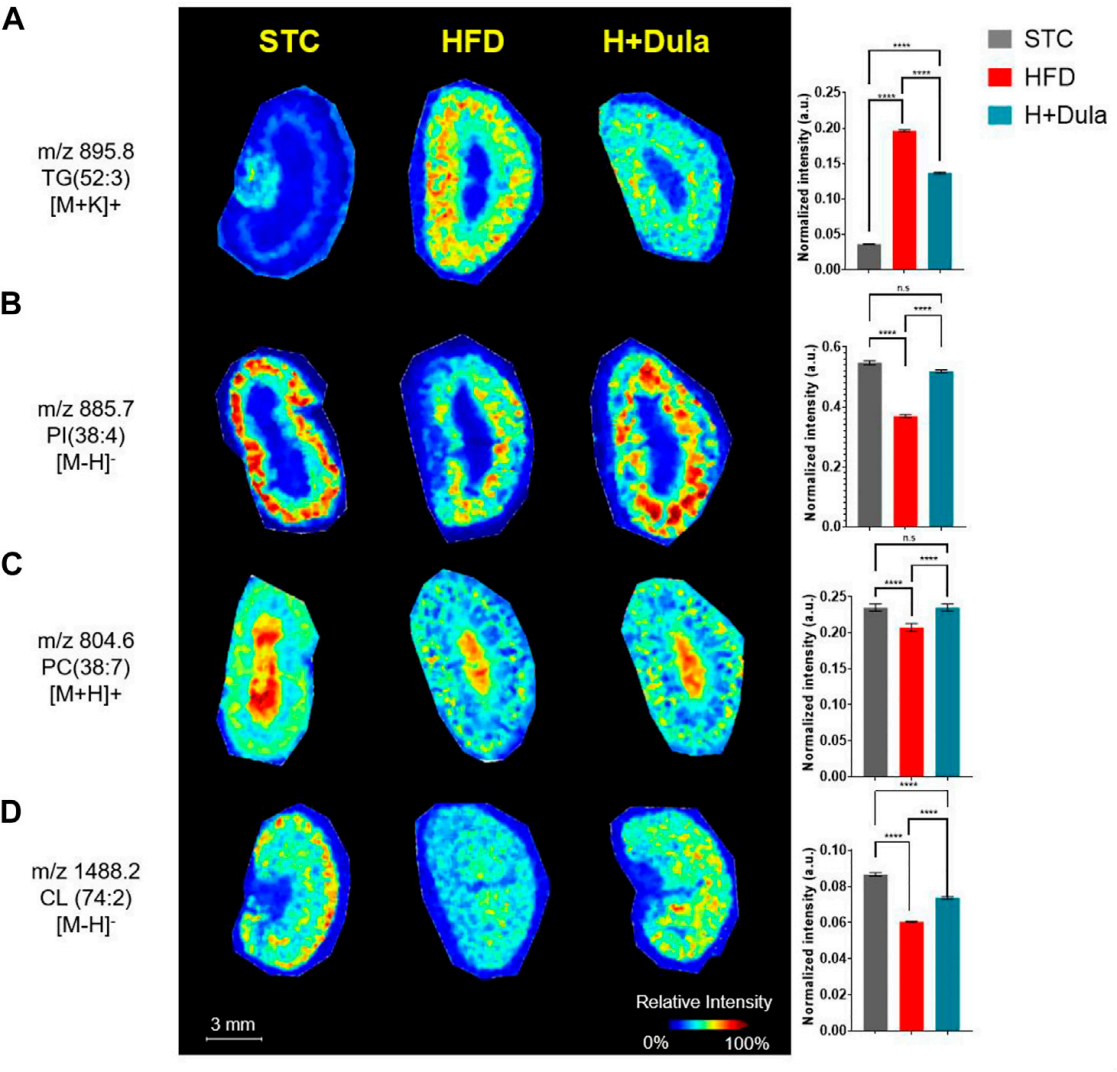
Dulaglutide can trigger the spatial redistribution of lipids in the kidneys of high-fat diet-fed mice visualized by MALDI-MSI. MALDI images of mouse kidney sections obtained at a spatial resolution of 100 *μ*m. Alterations in lipid content of PC, PI and CL in the kidney cortex after dulaglutide treatment are visualized by MALDI-MSI. Spatial visualization of **(A)** TG (52:3), [M + K]^+^, *m/z 895.8* was performed in positive mode. **(B)** PI (38:4), [M-H]^-^, *m/z* 885.7 was performed in negative mode. **(C)** PC(38:7), [M + H]^+^, *m/z* 804.6) was performed in positive mode and **(D)** CL (74:2), [M + H]^-^, *m/z* 1,488.2 was performed in negative mode. Bar chart of normalized intensities was plotted and used to calculate changes. Data represents means ± SEM, *n* = 6–9 mice per group. ****p < 0.0001 and n. s = not significant.

To explore the changes in the lipid species distribution, kidney cross sections were examined by MALDI-MSI. Consistent with our ORO staining and UHPLC/ESI-QTOF-MS results, high abundance of TG is observed, especially in the kidney cortex of high-fat diet-fed mice. For example, a low abundance of TG (52:3) (*m/z* = 895.8) was found in the outer medulla of the cortex region of the STC control by MALDI-MSI (Figure 4A, left panel). After high-fat diet treatment, TG (52:3) abundance increased dramatically in the cortex region and outer medulla of the HFD group (Figure 4A, middle panel). Decreased signal intensities of TG (52:3) due to dulaglutide treatment were observed mainly in the cortex region (Figure 4A, right panel).

Unexpectedly, a number of lipid species such as PI (38:4) (*m/z* = 885.7) and PC (38:7) (*m/z* = 804.6) decreased in abundance after the high-fat diet treatment (Figures 4B,C, left vs middle panels), and dulaglutide treatment almost restored their levels in the kidney cortex of the HFD group to the same level as that of STC group (Figures 4B,C). For cardiolipin, CL (74:2) (*m/z* = 1,488.2) was used as an example. A high abundance of CL (74:2) in the cortex region of mouse kidney of the STC control was observed as compared with that of the HFD group (Figure 4D, left vs middle panels). The reduction of CL (74:2) in the HFD group was ameliorated after dulaglutide treatment, especially for the cortex region (Figure 4D, middle vs right panels). In summary, both UHPLC/ESI-QTOF-MS and MALDI-MSI showed an induction in the abundance of cardiolipin, especially within the kidney cortex regions of the HFD group after dulaglutide treatment.

### Dulaglutide Increases the Level of Renal mtDNA Not via the Expression of Mitochondrial Biogenesis Genes

**FIGURE 5:**
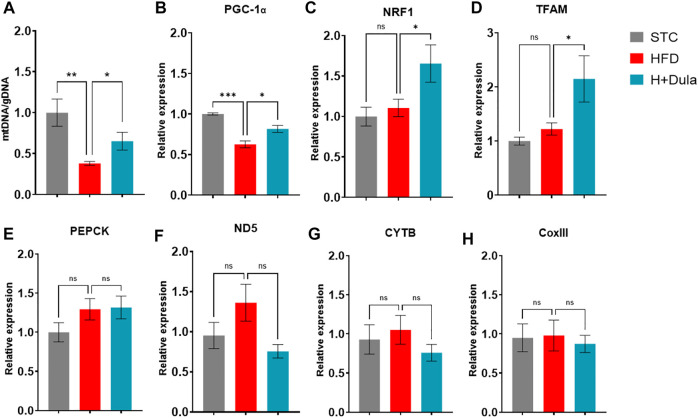
Dulaglutide treatment increases the level of renal mtDNA not via the expression of mitochondrial biogenesis genes. Quantification of **(A)** mitochondrial DNA (mtDNA) normalized with genomic DNA (gDNA) in renal tissue. RT-qPCR was used to determine the mRNA expression level of **(B)** PGC-1*α*, **(C)** Nuclear respiratory factor 1 (NRF1), **(D)** Mitochondrial transcription factor A (TFAM). **(E)** gluconeogenic phosphoenolpyruvate carboxykinase (PEPCK). **(F)** NADH-ubiquinone oxidoreductase chain five protein (ND5), **(G)** cytochrome b (CYTB), and **(H)** cytochrome oxidase III (CoxIII). Gene expression data were normalized against glyceraldehyde 3-phosphate dehydrogenase (GAPDH), and are shown relative to STC group, which were set arbitrary to 1. Values are mean ± SEM. *p < 0.05, **p < 0.01 and n. s = not significant.

As cardiolipin is a unique inner mitochondrial membrane lipid (Paradies et al., 2014), to explore whether the level of cardiolipin was associated with the number of mitochondria, we checked their amount of mitochondrial DNA (mtDNA) by RT-qPCR. In agreement with their cardiolipin levels, the STC group had the highest copy number of mtDNA (Figure 5A). High-fat diet treatment lowered the copy number of mtDNA in the kidney samples, while treatment of dulaglutide increased it (Figure 5A).

Previous studies demonstrated that GLP-1 increases mitochondrial biogenesis in various cell types via the expression level of the transcriptional coactivator PGC-1*α* (Li et al., 2011; Quan et al., 2020). We checked the expression level of PGC-1α and its regulated genes. Dulaglutide treatment increased the mRNA expression of PGC-1*α*-regulated genes NRF1 by only ∼30% (Figure 5C) and TFAM by only ∼50% (Figure 5D), but only ∼15% of mRNA expression of the PGC-1*α* (Figure 5B). There was no induction in the mRNA expression of PGC-1*α*–dependent gluconeogenic gene *PEPCK* (Figure 5E). The levels of mRNA expression of mitochondrial biogenesis genes ND5 complex I (Figure 5F), CYTB (complex III, Figure 5G), and COX III (complex IV, Figure 5H) were also unchanged. We further measured the protein expression of PGC-1*α*, and there was no change (Supplementary Figure S3). Thus, it is conceivable that dulaglutide’s increasing of the mtDNA copy numbers is not *via* mitochondrial biogenesis.

### Dulaglutide Treatment Increases the mRNA Expression of Cardiolipin Synthesis Genes

**FIGURE 6:**
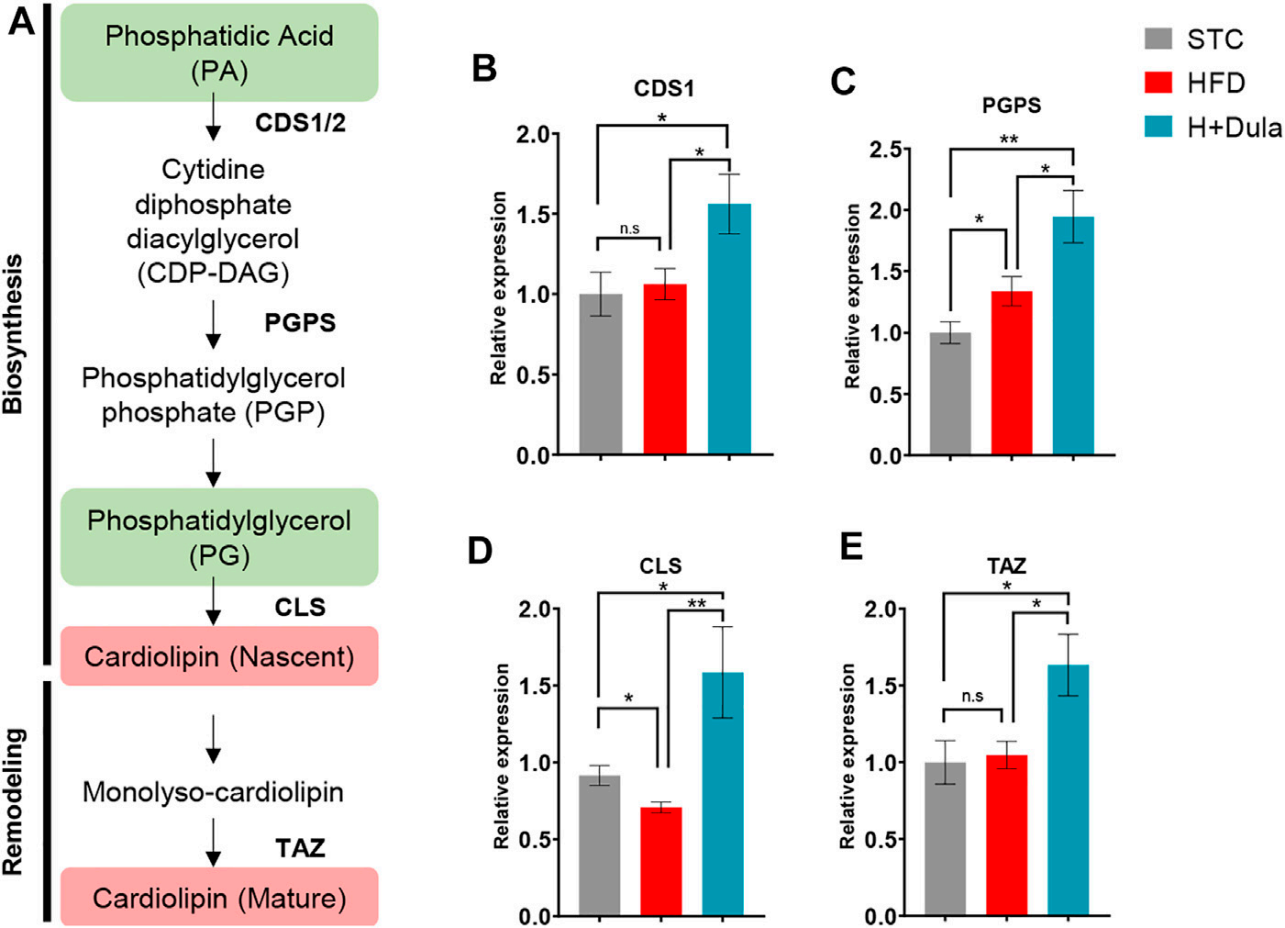
Dulaglutide treatment increases the mRNA expression of renal cardiolipin synthesis genes. **(A)** Overview of cardiolipin biosynthesis pathway. Lipid groups with increased and decreased abundance from UHPLC/ESI-QTOF-MS data are highlighted in red and green respectively. The mRNA level of enzymes involved in CL biosynthesis and remodeling includes **(B)** cytidine diphosphate diacylglycerol synthetase 1 (CDS1), **(C)** phosphatidylglycerol phosphate synthase (PGPS), **(D)** cardiolipin synthase (CLS) and **(E)** tafazzin (TAZ) by RT-qPCR. (Gene expression data were normalized against GAPDH, and are shown relative to STC group, which were set arbitrary to 1. Data represents means ± SEM, *n* = 6–9 mice per group. *p < 0.05, **p < 0.01 and n. s = not significant.

**FIGURE 7:**
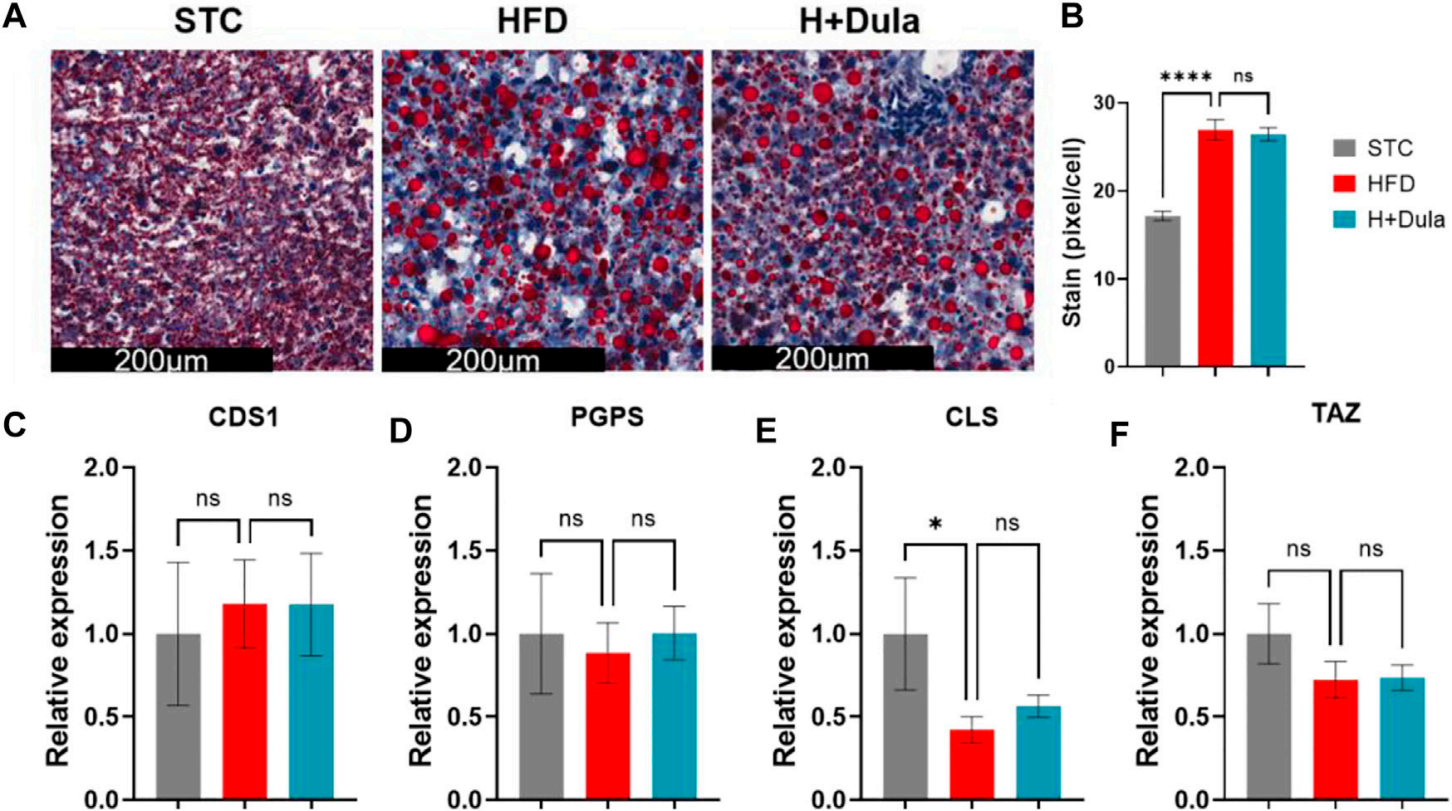
Dulaglutide cannot reduce ectopic lipid accumulation and increase the expression of cardiolipin synthesis genes in the livers of high-fat diet-fed mice. **(A)** The representative images of Oil Red O staining of liver from three different groups, *n* = 6–9 in each group. Image analysis was performed using ImageJ software, and the red stained pixel/cell was calculated for the different groups’ liver tissue sections. **(B)** The average red stained pixels were calculated from 30 random area from each mouse. **(C–F)** Dulaglutide treatment does not increase the mRNA expression of cardiolipin synthesis genes in liver. Gene expressions of enzymes involved in cardiolipin synthesis were quantified after dulaglutide treatment using reverse transcription polymerase chain reaction in the liver. The mRNA level of enzymes involved in CL biosynthesis and remodeling includes **(C)** cytidine diphosphate diacylglycerol synthetase 1 (CDS1), **(D)** phosphatidylglycerol phosphate synthase (PGPS), **(E)** cardiolipin synthase (CLS), and **(F)** tafazzin (TAZ) were measured by RT-qPCR. Gene expression data were normalized against GAPDH, and are shown relative to STC group, which were set arbitrary to 1. Data represents means ± SEM, *n* = 6–9 mice per group. ****p < 0.0001 and n. s = not significant.

As dulaglutide decreased cardiolipin precursors phosphatidic acid and phosphatidylglycerol levels (Table 1), we explored whether dulaglutide induced cardiolipin via the cardiolipin synthesis pathway (Figure 6A). We measured the mRNA expression of key cardiolipin synthesis enzymes (CDS1, PGPS, CLS, and TAZ). In brief, the mRNA expression of all four enzymes in H + Dula showed a significant increase as compared with that in the STC and HFD groups (Figures 6B–E). As GLP-1R is not expressed in hepatocytes (Pyke et al., 2014; Richards et al., 2014), we also checked the lipid accumulation and the expression of cardiolipin synthesis genes in their livers by ORO staining and RT-qPCR. Consistent with recent findings (Hupa-Breier et al., 2021), dulaglutide treatment cannot lower the hepatic lipid level of high-fat diet-fed mice (Figures 7A,B), and the expression level of hepatic cardiolipin synthesis genes remained unchanged as compared with HFD and H + Dula groups (Figures 7C–F). In summary, our data suggested that dulaglutide induces the expression of major enzymes in the cardiolipin synthesis pathway and hence enhances cardiolipin level in the kidney cortex of high-fed-diet-fed mice.

## Discussions

Lipids are one of the major energy sources of kidneys to maintain secretion and absorption. Under high-fat diet conditions, the excessive intake of energy enhances lipid synthesis, suppresses fatty acid oxidation, and hence promotes renal lipid accumulation (Stadler et al., 2015; Hirano, 2018; Thongnak et al., 2020). On one hand, the excess lipids repress the activation of energy-metabolism-related genes such as PGC1*α* and inhibit mitochondrial biosynthesis (Thongnak et al., 2020). On the other hand, excess lipids increase reactive oxygen species (ROS) production *via* overloading mitochondria by incomplete beta-oxidation (Thongnak et al., 2020). The overproduction of ROS in the kidney leads to renal inflammation, subsequently affecting renal injury, including albuminuria, glomerulosclerosis, and fibrosis (Jha et al., 2016). Excess nutrient influx into cells also upregulates a tubular-specific enzyme myo-inositol oxygenase (MIOX) accompanied by mitochondrial fragmentation and depolarization, releases of cytochrome c, and subsequent activation of apoptosis (Zhan et al., 2015).

In this study, we demonstrated that, similar to that in humans, treatment of dulaglutide can attenuate high-fat diet-induced renal dysfunction and morphological changes in mice. Dulaglutide does not simply improve the kidney function by lowering the overall lipid accumulation. Consistent with a previous study, our unbiased mass spectrometry approaches also identified that high-fat diet treatment lowered the level of cardiolipin in the kidneys of HFD-fed mice by imaging mass spectrometry (Hayasaka et al., 2016). Cardiolipin is a key lipid molecule that plays an essential role in the adequate structure of the mitochondrial cristae for optimal activity of mitochondrial respiratory complexes by correct assembly of supercomplexes (Paradies et al., 2014). Therefore, the level of cardiolipin is strongly correlated with mitochondrial efficiency in terms of increasing coupling efficiency for the synthesis of ATP and reducing ROS production (Szeto and Liu, 2018). In addition, cardiolipin and its oxidation products should be recognized as cellular signals that regulate various pathways, such as removal of dysfunctional mitochondria by mitophagy, execution of apoptosis, and activation of inflammasome (Dudek, 2017). A lowered renal cardiolipin level impairs kidney function via mitochondrial damage (Szeto, 2017). For example, anti-cardiolipin antibodies are important risk factors for acute renal graft dysfunction (Alchi et al., 2010). SS-31 (also named as elamipretide, MTP-131, or bendavia) is a member of the Szeto-Schiller (SS) peptide antioxidants known as selectively targeting inner mitochondrial membrane cardiolipin and hence preventing cardiolipin from converting cytochrome c into a peroxidase (Birk et al., 2013; Liu et al., 2014). A phase 2A clinical trial EVOLVE reported that percutaneous transluminal renal angioplasty adjunctive with SS-31 improved kidney function of patients with severe atherosclerotic renal artery stenosis in 2017 (Saad et al., 2017). The finding is supported by a recent observation that SS-31 can also protect db/db mice against progression of DKD, possibly by regulating cardiolipin remodeling (Miyamoto et al., 2020). These evidences support that cardiolipin is a promising therapeutic target for patients with renal diseases.

**FIGURE 8:**
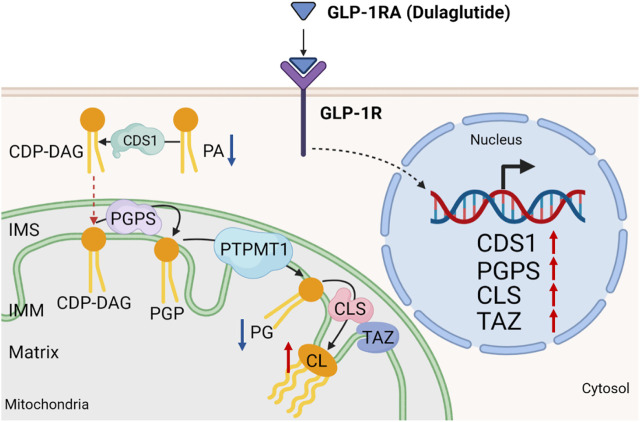
Working model. Dulaglutide attenuates high-fat diet-induced kidney damage by increasing cardiolipin level *via* the expression of cardiolipin synthesis genes. Activation of GLP-1R by dulaglutide led to the reduction in abundance of phosphatidic acids (PA) and phosphatidylglycerols (PG) and an increase in abundance of cardiolipins (CL) by increasing the expression of cardiolipin synthesis enzymes. CDS1, cytidine diphosphate diacylglycerol synthetase one; CLS, cardiolipin synthase; PGPS, phosphatidylglycerol phosphate synthase; PTPMT1, protein tyrosine phosphatase mitochondrial one; IMS, inner mitochondrial space; IMM, inner mitochondrial membrane. Red arrow ↑ denotes increased abundance and blue arrow ↓ denotes reduced abundance.

Instead of cardiolipin remodeling, we demonstrated that dulaglutide treatment can increase the level of cardiolipin in the kidney cortex region of high-fat diet-fed mice. Mechanistically, the increase in cardiolipin level by dulaglutide is mainly caused by the increase of the mRNA expression of major cardiolipin synthesis enzymes (Figure 8). The reason why dulaglutide preferentially increases cardiolipin distributed at the cortex region shall be due to the GLP-1R mainly expressing on the proximal tubular cells (Schlatter et al., 2007; Crajoinas et al., 2011). Approximately 80% of the filtrates, such as water, glucose, and salts, which pass through the glomerulus, are reabsorbed by proximal tubular cells via active transport (Bhargava and Schnellmann, 2017). To meet their energy needs, proximal tubular cells have more mitochondria than any other cells in the kidney to support filtrate transfer. In line with these observations, previous studies already demonstrated that GLP-1 modulates sodium and water homeostasis in the kidney most likely through a direct action via its GLP-1R in proximal tubular cells (Gutzwiller et al., 2006). Stimulation of GLP-1R also inhibits methylglyoxal-induced mitochondrial dysfunction (Nuamnaichati et al., 2020). Whether GLP-1 maintains mitochondrial activity for the active transport efficiency and protects mitochondria damaged by methylglyoxal via cardiolipin level remains to be explored.

A recent study using affinity pull-down and mass spectrometric analyses demonstrated that GLP-1 binds to mitochondrial trifunctional protein-*α* (MTP*α*) (Siraj et al., 2020). MTP*α* catalyzes the last three steps of mitochondrial beta-oxidation of long-chain fatty acids. The interaction inhibits MTP*α* and shifts the substrate utilization from oxygen-consuming fatty acid metabolism toward oxygen-sparing glycolysis and glucose oxidation (Siraj et al., 2020). Interestingly, MTP*α* also has a monolysocardiolipin acyltransferase activity that acylates monolysocardiolipin into cardiolipin (Taylor et al., 2012). It is interesting to further explore whether GLP-1 and its mimics can promote cardiolipin production via modulating the activity of MTP*α*.

Finally, to address the puzzle of our results on how dulaglutide increases the kidney’s mtDNA level without promoting mitochondrial biosynthesis, we paid attention to the fact that mtDNA locates in the mitochondrial matrix and is closely associated with the cristae (Glancy et al., 2020). mtDNA replication requires high coordination with the modulation of cristae structure, especially undergoing both mitochondrial fission and fusion events for maintaining the mitochondrial network structure (Chapman et al., 2020). A previous study demonstrated that dysregulation of the coordination will cause a loss of mtDNA integrity and copy number (Glancy et al., 2020). Therefore, lowering the cardiolipin levels can reduce mtDNA copy by formation of abnormal cristae structures (Chapman et al., 2020).

In conclusion, by utilizing UHPLC/ESI-QTOF-MS untargeted lipidomic approaches, we have found dulaglutide attenuates high-fat diet-induced kidney damage by increasing cardiolipin level via the expression of cardiolipin synthesis genes. As dulaglutide has circulating glucose-lowering and cardiolipin-upregulating effects in the kidney, dulaglutide is a potential ideal treatment regimen for DKD. We are looking forward to seeing the primary kidney outcome of the several ongoing randomized clinical trials of GLP-1RAs and exploring whether our proposed mechanism is conserved in mouse and human.

## Data Availability

The raw data supporting the conclusion of this article will be made available by the authors, without undue reservation.
