# Supplementary material for: Lipidomic Analysis Reveals the Protection Mechanism of GLP-1 Analogue Dulaglutide on High-Fat Diet-Induced Chronic Kidney Disease in Mice
Source: Front Pharmacol. 2022 Mar 1;12:777395. doi: 10.3389/fphar.2021.777395 (PMC8921774; doi:10.3389/fphar.2021.777395)
Supplement: Supplementary file 1 [file DataSheet1.PDF]

Lipidomic analysis reveals the protection mechanism of GLP-1 analogue Dulaglutide on high-fat diet-induced chronic kidney disease in mice

Martin Ho Yin Yeung[1], Ka Long Leung[1], Lai Yuen Choi[1], Jung Sun Yoo[1], Susan Yung[3], Pui-kin So[2] and Chi-Ming Wong[1,*]

Table of contents

Supplementary Figure S1……………………………………………………………….2

Supplementary Figure S2……………………………………………………………….3

Supplementary Table S1…..……………………….…………………………………….4

Supplementary Figure S1

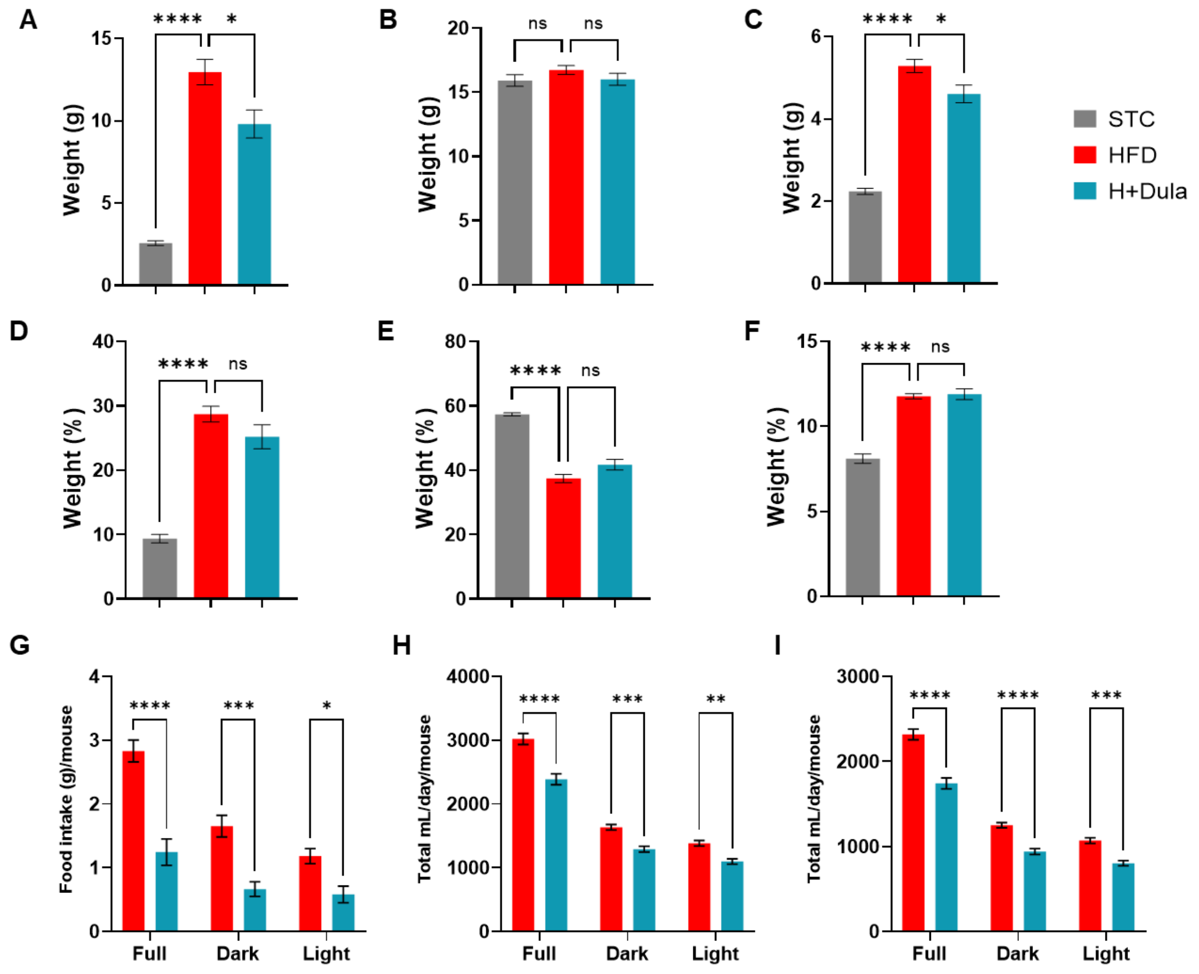

Supplementary Figure S1 Dulaglutide treatment lowers the fat mass of high-fat diet-fed mice. Mice were fed with standard chow or high-fat diet for 12 weeks. After dulaglutide treatment for 4 weeks, comparison of body composition was performed using ^{1}H nuclear magnetic resonance (^{1}H-NMR). Parameters including fat (A and D), lean body (B and E) masses and fluid (C and F) were measured by weight and as a percentage of overall body weight respectively. Mice were placed in individual cages for 24 hours to acclimatize prior to metabolic measurements for 24 hours food intake (G), volume of oxygen (H) and carbon dioxide (I). Data represents means ± SEM, n = 6 – 9 mice per group. * $P < 0.05$, **** $P < 0.0001$ and ns = not significant.

Supplementary Figure S2

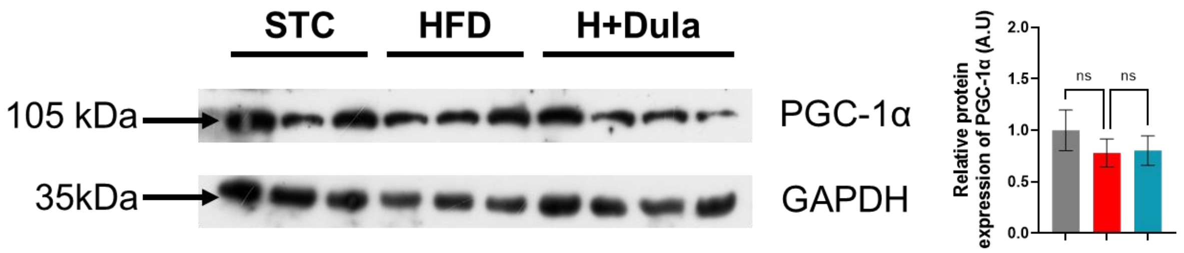

Supplementary Figure S2 Kidney PGC-1α protein expression level was determined by Western blotting (left). Each lane is a sample from a different individual. Quantification of kidney protein expression levels of PGC-1α were normalized to the expression of mouse GAPDH (right). STC group was set as 1 for fold-change calculation Data represent as mean ± SEM; n = 3 – 4 per group. ns = not significant.

Supplementary Table S1. Mouse primers for reverse transcription-quantitative polymerase chain reaction

NO.	Gene name	Forward	Reverse
1.	Cds1	TGTTCCCATATCAAGCGTCA	GGCTCACACTCTGTCACGAA
2.	Cls	ATCAGCTTTGGGAAGTGCTC	ACCTTGCTGATGAATGTTGGT
3.	CoxII (mtDNA)	TTTTCAGGCTTCACCCTAGATGA	GAAGAATGTTATGTTTACTCCTACGA ATATG
4.	CoxIII	CGTGAAGGAACCTACCAAGG	ATTCCTGTTGGAGGTCAGCA
5.	Cytb	CCCTAGCAATCGTTCACCTC	TCTGGGTCTCCTAGTATGTCTGG
6.	Gapdh	AGGTCGGTGTGAACGGATTTG	TGTAGACCATGTAGTTGAGGTCA
7.	mtnd5	ACCAGCATTCCAGTCCTCAC	ATGGGTGTAATGCGGTGAAT
8.	Nrf1	GCACCTTTGGAGAATGTGGT	GGGTCATTTTGTCCACAGAGA
9.	Pepck	AGTGCCCATCCCCAAAACT	CACCACATAGGGCGAGTCTG
10.	Pgc-1α	AAACTTGCTAGCGGTCCTCA	TGGCTGGTGCCAGTAAGAG
11.	Pgps	CGACCTCAAGGTCTCCATTC	GTTTGCACCACTCAGGATGA
12.	Taz	CTGGGGGATCCTAAAACTCC	AGCGCAGGAACTCAGAACTC
13.	Tfam	CCTTCGATTTTCCACAGAACA	GCTCACAGCTTCTTTGTATGCTT
14.	β-globin (gDNA)	GAAGCGATTCTAGGGAGCAG	GGAGCAGCGATTCTGAGTAGA
